# DNA methylation in glioma: clinical biomarkers and emerging biosensor-based diagnostics

**DOI:** 10.3389/fonc.2026.1965742

**Published:** 2026-10-19

**Authors:** Abdullah Jabri, Shamsa Hilal Saleh, Mohamed Alsharif, Abdulfatah Hamouch, Juman Alammar, Hamidreza Emadzadeh, Raja Chinnappan, Ahmed Yaqinuddin, Mohammed Imran Khan

**Affiliations:** 1 College of Medicine, Alfaisal University, Riyadh, Saudi Arabia; 2 King Faisal Specialist Hospital and Research Centre, Riyadh, Saudi Arabia; 3 Faculty of Medicine, Wroclaw Medical University, Wrocław, Poland; 4 School of Medicine, St. George’s University, St. George’s, Grenada; 5 King Faisal Specialist Hospital and Research Center, Jeddah, Saudi Arabia

**Keywords:** biosensors, circulating tumor DNA, DNA methylation, glioblastoma, glioma

## Abstract

Gliomas are the most common malignant primary tumors of the central nervous system (CNS) and are characterized by marked molecular heterogeneity, aggressive clinical behavior, and poor patient outcomes. Among the epigenetic alterations implicated in glioma pathogenesis, DNA methylation has emerged as a key regulatory mechanism with important diagnostic, prognostic, and predictive value. Clinically established biomarkers, including O6-methylguanine-DNA methyltransferase (MGMT) promoter methylation and the glioma CpG island methylator phenotype (G-CIMP), have transformed patient stratification and therapeutic decision-making, while additional methylation signatures continue to expand the molecular classification of glioma. Conventional methylation detection methods, although highly accurate, remain limited by their cost, complexity, and dependence on specialized laboratory infrastructure. This review summarizes current knowledge of DNA methylation biology in glioma, discusses established and emerging methylation biomarkers, and critically examines recent advances in biosensor-based detection technologies, including electrochemical, optical, microfluidic, and nanopore-based platforms, together with enzyme-assisted and immunoaffinity-based molecular recognition strategies that can be combined with these transducers. These technologies, particularly when combined with nanomaterials, signal amplification strategies, and liquid biopsy approaches, offer highly sensitive, rapid, and minimally invasive detection of methylated circulating tumor DNA, supporting the development of point-of-care diagnostics. Despite encouraging progress, challenges related to analytical standardization, tumor heterogeneity, cross-reactivity, large-scale clinical validation, and regulatory translation remain significant barriers to routine implementation. By integrating recent advances in glioma epigenetics with emerging biosensing technologies, this review provides a comprehensive overview of the current landscape and highlights future opportunities for translating DNA methylation biomarkers into clinically accessible precision diagnostic platforms.

## 1 Introduction

Gliomas constitute the most common malignant and lethal primary brain tumors in adults (1, 2). Their clinical intractability reflects, in part, profound molecular heterogeneity that conventional histopathology cannot fully capture. Epigenetic alterations are now understood to be central to this heterogeneity, and among them, aberrant DNA methylation, the covalent addition of methyl groups to cytosine residues at CpG dinucleotides, stands out for both its mechanistic importance and its translational accessibility (3). The enzymes responsible, DNMT1, DNMT3A, and DNMT3B, are frequently dysregulated in glioma, producing widespread changes in gene expression, chromatin architecture, and tumor cell identity.

One of the most consequential advances in neuro-oncology over the past decade has been the recognition that genome-wide DNA methylation profiles constitute reliable molecular fingerprints for CNS tumor classification (3, 4). The Heidelberg classifier is now embedded in neuropathological practice and is formally endorsed by the 2021 WHO Classification of CNS Tumors (WHO CNS5), which incorporates methylation profiling alongside histological and molecular criteria as a diagnostic criterion for defined tumor types (3–6). Spatial studies have shown that methylation class assignments can remain concordant across anatomically distinct tumor regions, supporting the use of single-biopsy methylation profiling for classification in many cases. However, this concordance is not uniform across all gliomas, and intratumoral methylation heterogeneity may limit the ability of a single biopsy to fully capture the epigenetic diversity of some tumors (7).

Several specific methylation biomarkers have moved beyond classification into direct clinical use. Promoter methylation of MGMT silences the gene, impairing repair of temozolomide-induced alkylation damage, and predicts survival benefit from chemotherapy in newly diagnosed glioblastoma (GBM), a finding first established by Hegi et al. and subsequently confirmed across independent cohorts and meta-analyses (2, 8). The glioma CpG island methylator phenotype (G-CIMP) arises from isocitrate dehydrogenase (IDH) mutation-derived 2-hydroxyglutarate, which inhibits TET dioxygenases and produces widespread CpG island hypermethylation; G-CIMP high/G-CIMP low reliably identifies IDH-mutant tumors and independently confers a favorable prognosis (4, 5). At the level of individual loci, promoter hypomethylation of CAMKK2 drives its overexpression, promoting invasion and independently predicting poorer survival (9), while methylation-mediated silencing of miR-133a sustains pro-tumorigenic signaling through PPAR-gamma, whose effects are context-dependent, and methylation-mediated silencing of miR-410 promotes glioma growth through the STAT3 pathway (10, 11).

Methylation profiling also captures biologically meaningful variation in the tumor immune microenvironment. Large-scale analyses have shown that gliomas segregate into immune-excluded, immune-desert, and immune-inflamed subtypes based on methylation regulatory patterns, with the latter associated with PD-L1 expression and responsiveness to immune checkpoint blockade (12). Epigenetic heterogeneity, whether measured as the proportion of intermediately methylated sites or as single-cell DNAme disorder, independently predicts shorter time to recurrence and worse survival in IDH-wildtype GBM (13). Non-invasive liquid biopsy approaches have extended these insights to the clinical monitoring setting: the glioma epigenetic liquid biopsy (GeLB) score, a serum methylation signature, has demonstrated utility in distinguishing true progression from pseudo-progression; refer to section 3.1 for more details. In this review, cell-free DNA (cfDNA) signifies the complete collection of DNA fragments found in blood or other bodily fluids, sourced from both tumor and non-tumor cells, whereas circulating tumor DNA (ctDNA) specifically refers to the tumor-derived portion of cfDNA. Due to the limitations of current assays in isolating the tumor-derived fraction, methylation signatures like the GeLB score are more accurately referred to as cfDNA-based, where tumor-associated signals are inferred instead of directly isolated, whereas platforms specifically designed to enrich or detect tumor-specific mutations or methylation patterns are termed ctDNA-targeted.

Artificial intelligence has further extended the reach of methylation diagnostics. Explainable AI frameworks applied to the Heidelberg classifier confirm that its decisions rest on functionally meaningful regions, including CpG islands and enhancers, while imaging-based models have shown that methylation class can be estimated from multimodal MRI data, raising the prospect of pre-surgical molecular stratification without tissue sampling (14, 15). Despite these advances, standard methylation detection methods, including bisulfite sequencing, methylation-specific PCR, and array-based platforms, remain costly, technically demanding, and poorly suited to point-of-care settings. Biosensor-based platforms, spanning electrochemical, optical, and microfluidic designs, have emerged as promising alternatives that offer speed, miniaturization, and sensitivity compatible with near-patient use. Unlike previous reviews that have primarily examined the biological and clinical significance of DNA methylation in glioma or biosensor technologies more broadly, this review integrates these areas by linking established and emerging glioma methylation biomarkers with biosensor platforms for their detection. Emphasis is placed on analytical performance, glioma-specific validation, limitations, and translational potential, with the aim of identifying the key challenges that must be addressed to advance methylation-based biosensors toward clinical application.

An overview of the key concepts discussed in this review, including the role of DNA methylation in glioma and emerging biosensor-based diagnostic approaches, is illustrated in **Figure 1**.

**Figure 1:**
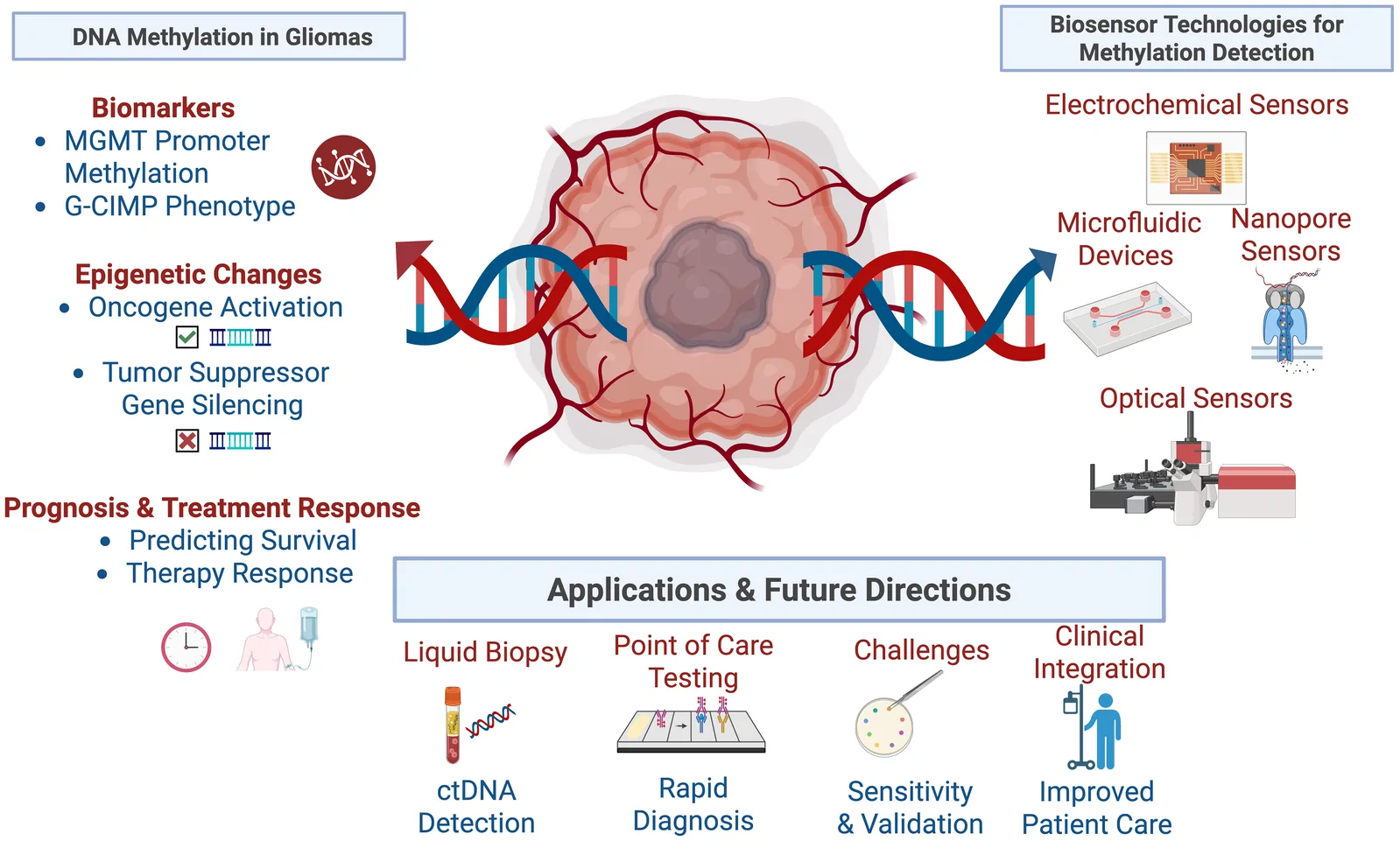
DNA methylation biology, biomarkers, biosensor technologies, and clinical translation in glioma. Schematic overview integrating the major concepts discussed in this review. DNA methylation alterations are shown in relation to glioma biology, including changes in gene regulation, tumor phenotype, and molecular classification. Established and emerging methylation biomarkers, including MGMT promoter methylation, the glioma-CpG island methylator phenotype (G-CIMP), IDH-associated methylation signatures, and circulating DNA-based methylation signatures, are linked to their diagnostic, prognostic, predictive, and monitoring applications. The figure also summarizes emerging biosensor approaches, including electrochemical, optical, microfluidic, and nanopore-based platforms, for detecting methylation-associated DNA signals. Current challenges, including analytical sensitivity, biological heterogeneity, standardization, clinical validation, and regulatory translation, are shown alongside the potential for future point-of-care (POC) and near-patient applications. The figure is intended as an integrated overview linking glioma methylation biology and biomarkers with emerging detection technologies and their potential clinical translation.

## 2 DNA methylation in glioma biology

### 2.1 Genome-wide methylation patterns in glioma

Genome-wide DNA methylation profiling has changed glioma classification by revealing unique epigenetic structures that often outperform standard histology in diagnostic precision (4). The glioma CpG island methylator phenotype (G-CIMP), a marked global hypermethylation seen in gliomas with isocitrate dehydrogenase mutations, is the best example of this (4). In addition, the subset of IDH-wildtype GBMs can also exhibit partial CpG island methylation. Minimal CpG panels can also achieve diagnostic accuracy, as shown by an 11-site epi-signature that distinguishes IDH1/IDH2-mutant from wild-type tumors (16). This epigenetic heterogeneity is both intratumoral and intertumoral. Nearly 40% of glioblastomas have diverse methylation subtypes within a single tumor, according to systematic multi-region sampling studies (5). However, the degree of heterogeneity varies by subtype; IDH-wildtype GBM and high-grade meningiomas exhibit significant geographic heterogeneity, whereas IDH-mutant gliomas show very uniform methylation-based classifications (17). Therefore, although single-biopsy methylation profiling can provide reliable classification in many cases, intratumoral heterogeneity should be considered when interpreting methylation profiles from individual tumor samples. Our understanding of the biology of gliomas has been further refined by mapping different epigenetic signatures, such as enhanced proliferation, inflammation, and loss of synaptic transmission, to certain glioma archetypes even within these complex landscapes (18).

### 2.2 Specific genes and pathways affected by aberrant methylation

The majority of glioma pathophysiology is scientifically based on aberrant DNA methylation, which directly affects the expression of particular oncogenes, tumor suppressors, and DNA repair genes. Methylation-dependent silencing of the O^6^-methylguanine-DNA methyltransferase (MGMT) gene is the most clinically significant example. A key component of GBM patient categorization is MGMT promoter methylation, a potent predictive biomarker for reaction to the alkylating drug temozolomide (TMZ) (19). Because of its significance, new methods are being developed to find additional methylation-based predictors of TMZ response, even in tumors that have an unmethylated MGMT promoter (20). Beyond MGMT, certain oncogenic factors influence and interact with methylation patterns. A genetic driver can systematically remodel the epigenetic landscape, as demonstrated by the unique methylome of EGFR-amplified glioblastomas, which is defined by the hypomethylation of pathways controlling DNA replication, packaging, and gene silencing (21). Similarly, it has been demonstrated that the epigenetic regulator ICBP90 accelerates glioma development by binding to and maintaining the promoter methylation of the tumor suppressor DKK3, which silences its expression, promotes carcinogenesis, and decreases sensitivity to platinum-based treatment (22). Replication repair-deficient (RRD) high-grade gliomas, by contrast, exhibit a distinct genome-wide promoter hypomethylation phenotype and a hypermutant genotype resulting from germline defects in DNA replication repair pathways. Unlike the widespread CpG island hypermethylation observed in G-CIMP-high gliomas, these tumors display extensive promoter hypomethylation, particularly affecting genes involved in cell-cycle regulation and metabolism (23).

### 2.3 Functional consequences of DNA methylation alterations

Glioma cell behavior is significantly and directly impacted by methylation-driven changes in gene expression. The nuclear pore complex protein NUP37 promotes glioma cell invasion and proliferation by recruiting the maintenance methyltransferase DNMT1 to silence tumor-suppressor gene promoters; consistent with this pro-tumorigenic mechanism, DNMT1 overexpression rescues the growth-suppressive phenotype caused by NUP37 depletion, confirming that NUP37 acts upstream of DNMT1-mediated epigenetic silencing (24). Moreover, it appears that the DNA repair protein MGMT itself plays a more extensive role in tumor pathogenesis. A fundamental invasion-proliferation switch has been linked to a canonical chemotherapy-resistance mechanism through an inverse relationship in which MGMT expression positively correlates with proliferation, and its silencing via promoter methylation is significantly associated with increased expression of the invasion marker SPARC (25). Another important factor influencing treatment resistance is this junction with DNA repair mechanisms. DNA methylation-dependent mechanisms of chemoresistance and radiation resistance are present in glioma stem-like cells (GSCs), which are major contributors to treatment failure (19). One such method is the methylation of the CDKN2A promoter, which confers GSCs with particular resistance to carmustine and increases their potential for self-renewal, demonstrating that methylation of cell-cycle regulators directly causes stem-cell-driven drug resistance (26).

### 2.4 Relationship between DNA methylation and other molecular alterations in glioma

DNA methylation is intricately linked to a complex web of chemical changes rather than functioning in a vacuum. With copy number variations (CNVs), this crosstalk is very noticeable. Phylogenetic analyses indicate that events like CDKN2A/B loss may occur early in tumor development, and intratumoral methylation heterogeneity in GBM frequently co-occurs with CNV heterogeneity in important genes, including CDKN2A/B, EGFR, and PTEN (5). CNVs, including diagnostically important events such as 1p19q co-deletion, can also be inferred concurrently from DNA methylation array data (16). MicroRNAs (miRNAs) are an additional component of the regulatory network. Four glioma subtypes with different prognoses and immune-related traits were identified by a systematic integrative multi-omics analysis that found that the expression of 719 miRNAs is linked to changes in either CNV or promoter methylation (27).Immune evasion depends on the interaction of methylation, CNV, and miRNA expression, which may have important consequences for patient stratification in immunotherapy. The deep, systematic link between genotype, epigenotype, and tumor physiology is highlighted by the strong predictive ability of these integrated patterns, such that machine learning models can now reliably predict mutation status and methylation class from MRI data (28).

## 3 DNA methylation as a biomarker for glioma

### 3.1 Diagnostic biomarkers

DNA methylation profiling has become integral to the molecular classification of gliomas, functioning alongside conventional histopathology to deliver substantially more precise diagnostic assignments. This shift is codified in the 2021 WHO Classification of CNS Tumors (WHO CNS5), which formally incorporates molecular markers, including methylation profiling, as a robust complementary approach to classifying brain tumors (5, 6). Genome-wide methylation analysis resolves biologically and clinically distinct subgroups that histology alone cannot reliably differentiate. Large studies have demonstrated that glioma patients stratify into molecular subtypes corresponding to distinct immune phenotypes, including immune-excluded, immune-desert, and immune-inflamed, that are reproducible across independent datasets (12).

A central component of diagnostic methylation profiling is the determination of IDH mutation status, the primary oncogenetic driver in adult-type diffuse gliomas. IDH mutations produce widespread promoter CpG island hypermethylation, known as the glioma CpG island methylator phenotype (G-CIMP), which serves as an epigenetic surrogate for IDH status (5). A refined diagnostic approach employing an 11-CpG epi-signature targeting genes including *CUL7*, *TRANK1*, *SNCB*, and *MECOM* demonstrated high accuracy in distinguishing IDH-mutant from IDH-wildtype gliomas, including detection of rare IDH1 and IDH2 variants that immunohistochemistry alone would miss (16). The G-CIMP-high profile is almost invariably associated with IDH-mutant status and a favorable prognosis, while G-CIMP-low tumors exhibit more aggressive biological behavior (29).

Complementing tissue-based diagnostics, non-invasive liquid biopsies have demonstrated growing clinical utility. The glioma epigenetic liquid biopsy (GeLB) score, derived from a serum cell-free DNA methylation signature, achieved 100% sensitivity and 97.78% specificity for glioma detection in an independent validation cohort; the serum methylome corresponded closely to matched primary glioma tissue methylomes (30). The Heidelberg methylation classifier can be applied to both fresh and formalin-fixed, paraffin-embedded tumor samples to assign clinically meaningful methylation classes, including RTK1, RTK2, and mesenchymal subgroups, that refine GBM diagnosis (31). Methylation arrays additionally yield copy-number variation data, enabling identification of structural alterations such as EGFR amplification, chromosome 7 gain, and chromosome 10 loss, features that, in combination with TERT promoter mutation, support a definitive IDH-wildtype GBM diagnosis (31). This performance was detailed in one independent validation group by Sabedot et al; to consider this sensitivity as generalizable, replication in larger, independent, multi-institutional groups is required, and the flawless sensitivity in any single group should be interpreted cautiously due to possible cohort-selection biases (30).

### 3.2 Prognostic biomarkers

Accumulating evidence establishes DNA methylation markers as powerful prognostic tools that refine patient risk stratification and inform therapeutic decision-making. MGMT promoter methylation is the most extensively validated prognostic biomarker in glioblastoma. A meta-analysis of 14 studies including 1,231 patients reported that patients with an unmethylated MGMT promoter had a significantly higher risk of death than those with methylated tumors (HR 1.66, 95% CI 1.32–2.09; P < 0.0001), confirming the favorable prognostic impact of MGMT promoter methylation (32). In the EORTC-26101 trial for progressive GBM, MGMT promoter methylation remained strongly prognostic for both overall survival and progression-free survival, with methylated patients achieving a median OS of 11.5 months compared to 7.8 months in unmethylated patients (P < 0.0001) (31). This trial also demonstrated that MGMT promoter methylation retained its prognostic value across patients treated with lomustine alone or lomustine plus bevacizumab, indicating its relevance in bevacizumab-containing treatment regimens (31).

Methylation-based classification systems provide independent prognostic resolution beyond individual markers. In the CATNON trial, a prospective phase III trial of patients with 1p/19q non-co-deleted anaplastic glioma, the CNS tumor classifier identified A_IDH_HG tumors (high-grade methylation class) as carrying significantly worse overall survival than A_IDH lower-grade tumors (HR 2.43, 95% CI 1.73 to 3.40, P < 0.0001) (29). The glioma classifier demonstrated that G-CIMP-low tumors had markedly inferior prognosis compared to G-CIMP-high tumors (HR 4.12, 95% CI 2.37 to 7.18, P < 0.0001), with further stratification possible using a seven-CpG progression-risk score that identified risk versus no-risk G-CIMP-high tumors (HR 1.64, P = 0.01) (29). In progressive GBM from the EORTC-26101 cohort, the RTK1 methylation phenotype was associated with a median OS of 7.6 months, compared to 9.2 months for RTK2 and 10.5 months for mesenchymal tumors (P = 0.024), an effect that remained significant in multivariate analysis independent of MGMT status (31).

Quantitative methylation scores capture broader epigenetic instability with prognostic utility. The DNA methylation score (DMS), derived from nearly 5,700 prognosis-related differentially expressed genes, functions as an independent prognostic marker: low DMS correlated with shorter overall survival across all glioma grades (P < 0.001) (12). The proportion of sites with intermediate methylation (PIM score), a measure of DNA methylation heterogeneity, showed that higher PIM was associated with worse outcomes, while lower PIM correlated with better survival and slower disease progression (13). At the single-cell level, elevated DNA methylation disorder, particularly increasing promoter DNAme disorder between primary and recurrent IDH-wildtype gliomas, predicted shorter time to second surgery (univariate log-rank P = 0.04; multivariate HR 1.55, P = 0.03) (33). Collectively, these findings demonstrate that both absolute methylation levels and patterns of epigenetic heterogeneity convey independent prognostic information.

### 3.3 Predictive biomarkers

The most established predictive application of DNA methylation in glioma remains MGMT promoter methylation, which identifies patients with GBM who benefit from TMZ chemotherapy (31, 34). This predictive value extends to lomustine and other alkylating agents; dual *MGMT* inactivation via both promoter hypermethylation and loss of chromosome 10q confers heightened sensitivity to TMZ (34). Although Section 3.2 identifies MGMT methylation as a prognostic factor independent of treatment, its predictive significance specifically relates to the additional advantage from TMZ/alkylating therapy, a difference reinforced by randomized trial evidence (EORTC-26101) compared to retrospective cohort/meta-analytic prognostic insights by Sareen et al. (32). In the EORTC-26101 trial, MGMT promoter methylation demonstrated predictive relevance in patients treated with lomustine alone or in combination with bevacizumab (31).

Mutation-methylation interactions have yielded additional clinically actionable predictive insights. In the EORTC-26101 cohort, *NF1* mutations, which are common in IDH-wildtype GBM, were identified as predictive of response to bevacizumab: patients with *NF1*-mutant tumors receiving lomustine plus bevacizumab survived significantly longer than those receiving lomustine alone (P = 0.0046), with no corresponding benefit observed in *NF1* wild-type patients (31).

DNA methylation signatures also inform the likelihood of response to immune checkpoint blockade. The high-DMS subgroup, characterized by elevated expression of immune-related genes and higher PD-L1 levels, was associated with better response to anti-PD-1/PD-L1 immunotherapy in two independent cohorts (12). The GeLB score demonstrated predictive utility beyond diagnosis: it decreased in patients responding to treatment, increased with disease progression, and distinguished true progression from pseudo progression by remaining elevated in true progression while falling below the diagnostic threshold in treatment-induced necrosis (30).

Methylation-driven silencing of tumor-suppressor microRNAs represents an additional therapeutic target. The DNMT inhibitor 5-azacytidine reverses promoter methylation of *miR-133a*, restoring its expression and inducing anti-proliferative and pro-apoptotic effects in glioma cells through PPAR-gamma targeting, illustrating the mechanistic rationale for epigenetic priming strategies (10).

The tumor immune microenvironment is systematically shaped by methylation patterns and may itself function as a predictive biomarker. The immune-inflamed methylation pattern (Pattern C) is enriched for activated CD8+ T cells and dendritic cells, which are hallmarks of an immunotherapy-responsive microenvironment, whereas the immune-desert pattern (Pattern B) correlates with stromal activation and immune exclusion, both associated with resistance (12).A risk score derived from eight CpG sites linked to distinct immune cell populations (the CMHR score) achieved high accuracy in discriminating IDH mutation status (AUC 0.96) and positively correlated with cytotoxic T-lymphocyte infiltration in the tumor microenvironment (13). These findings collectively highlight the evolving role of DNA methylation biomarkers as predictors of response across chemotherapy, targeted therapy, and immunotherapy, a paradigm shift that is reshaping therapeutic decision-making in neuro-oncology. Key DNA methylation biomarkers and their clinical applications are summarized in **Table 1**.

**Table 1:** Summary of major DNA methylation biomarkers in glioma: methylation patterns, biological roles, and clinical utility.

Biomarker	Methylation pattern	Biological role	Clinical utility	Key references
MGMT promoter	Hypermethylation causing gene silencing	Suppresses DNA alkylation repair; linked to invasion-proliferation switch	Predictive: TMZ and alkylator response. Prognostic: improved OS in GBM	(2, 31, 32, 34)
G-CIMP (IDH-associated)	Global CpG island hypermethylation	Driven by 2-HG inhibition of TET enzymes; widespread transcriptional silencing	Diagnostic: IDH surrogate marker. Prognostic: G-CIMP-high associated with favorable prognosis	(4, 29)
11-CpG IDH epi-signature	Selective locus hypermethylation	Distinguishes IDH-mutant from IDH-wildtype tumors at high accuracy	Diagnostic: IDH classification including rare variants missed by immunohistochemistry	(16)
GeLB Score	Serum cell-free DNA methylation	Serum methylome reflects primary tumor epigenome	Diagnostic via liquid biopsy. Monitoring: tracks treatment response and progression	(30)
DMS (DNA methylation score)	Multi-locus quantitative score	Integrates approximately 5,700 prognosis-related gene methylation states	Prognostic: independent predictor of OS across all grades. Predictive of immunotherapy response	(12)
CMHR score (8-CpG)	Immune-cell-associated CpG sites	Reflects tumor immune microenvironment composition	Predictive: IDH status discrimination (AUC 0.96); correlates with cytotoxic T-lymphocyte infiltration	(13)
RTK1/RTK2/Mesenchymal class	Genome-wide methylation class assignment	Reflects distinct GBM biological subtypes	Prognostic: RTK1 worst OS (7.6 months), mesenchymal best (10.5 months); guides clinical trial stratification	(31)

(**Table 3**, which maps these biomarkers to biosensor strategies, is presented within Section 4.5.).

## 4 Biosensors for point-of-care glioma diagnostics

The development of biosensor-based platforms for rapid, point-of-care (POC) diagnostics has been prompted by the growing recognition of DNA methylation as a clinically significant biomarker in gliomas. Despite their high analytical sensitivity, conventional methods such as bisulfite sequencing and methylation-specific PCR are constrained by relatively complex workflows and the need for specialized laboratory equipment. Biosensors are promising tools for decentralized glioma diagnosis, offering potential advantages including portability, shorter assay times, reduced sample requirements, and simplified workflows (35, 36). The main biosensor platforms used for DNA methylation detection, including electrochemical, optical, microfluidic, and nanopore-based systems, are summarized in **Figure 2** and **Table 2**. However, the platforms outlined below differ significantly in their degree of glioma-specific validation; refer to Section 5.6 for a summarized discussion of this issue across all platforms.

**Figure 2:**
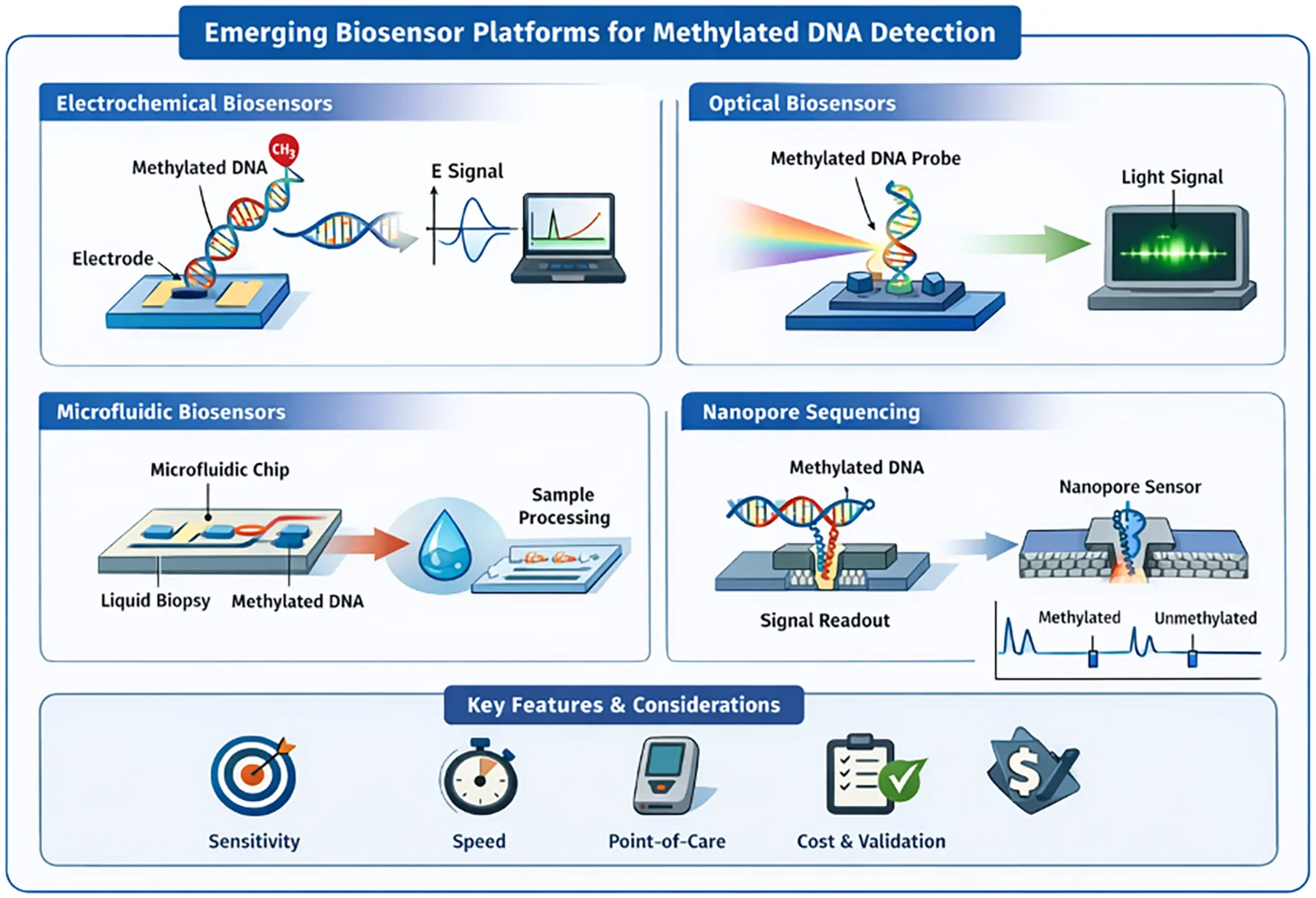
Biosensor platforms for DNA methylation detection. Schematic representation of the principal biosensor technologies discussed for detection of DNA methylation biomarkers. Electrochemical biosensors detect methylation-associated changes through electrical signals generated at functionalized electrodes and offer advantages in sensitivity, miniaturization, multiplexing, and portability. Optical biosensors use optical signal transduction, including fluorescence, colorimetric, surface plasmon resonance (SPR), or surface-enhanced Raman scattering (SERS)-based approaches, to detect methylation-associated molecular interactions. Microfluidic platforms integrate sample handling and biosensing within miniaturized devices, potentially reducing sample volume, processing time, and reagent requirements. Nanopore-based approaches enable detection of DNA methylation through changes in ionic current and can provide single-molecule information. These platforms represent different stages of technological development, and their potential for rapid or point-of-care (POC) use varies according to assay complexity, sample preparation requirements, instrumentation, and degree of clinical validation.

**Table 2:** Comparison of biosensor platforms for DNA methylation detection in glioma.

Biosensor type	Detection principle	Advantages	Limitations	Sample throughput	Cost per test (relative)	Analytical performance	Current clinical status	Evidence and potential relevance to glioma
Electrochemical	Measures electrical changes (current, voltage, impedance) upon binding of methylated DNA	High sensitivity, portable, easy miniaturization	Susceptible to signal interference; requires electrode optimization	Low–moderate (single-electrode); higher with screen-printed multiplexed arrays	Low (simple instrumentation, low reagent cost)	0.96 aM (Chen et al., 2019, synthetic DNA, HCR + tetrahedral DNA nanostructure); femtomolar–attomolar range typical across recent reviews. Sub-attomolar LOD reported in proof-of-concept methylated-DNA assays; clinical glioma validation lacking.	Early clinical evaluation	Primarily demonstrated in general DNA-methylation assays; direct validation using glioma-specific targets or clinical samples remains limited. Potentially applicable to MGMT and other glioma-associated methylation biomarkers.
Optical (Fluorescence, SPR, SERS)	Detects changes in fluorescence, absorbance, surface plasmon resonance, or Raman scattering	High accuracy, label-free options, real-time monitoring	Expensive equipment; limited portability	Moderate; multiplexing feasible with multicolor fluorophores	Moderate–high	1 zM reported LLOQ (Adampourezare et al., 2021, synthetic target) — theoretical/idealized; real-sample LOD likely far higher due to stochastic sampling limits at these concentrations. Reported ultralow analytical concentrations in synthetic/model DNA systems; clinical-sample performance not established	Laboratory validation	Mostly supported by proof-of-concept DNA-methylation studies using model or non-glioma targets. Direct clinical validation for glioma methylation biomarkers remains limited.
Microfluidic-based	Integrates fluid handling with biosensing on miniaturized chips	Low sample volume, rapid processing, automation	Fabrication complexity; integration challenges	High (designed for rapid, low-volume, potentially multiplexed processing)	Low-Moderate	High analytical performance with rapid sample processing	Prototype and pilot studies	Potentially suitable for glioma liquid-biopsy workflows, but clinical validation for glioma-specific methylation detection remains limited.
Nanopore-based	Detects ionic current changes as single DNA molecules pass through nanopores	Single-molecule resolution, real-time sequencing, label-free	High technical complexity; demanding data analysis	Moderate–high (multiplexed barcoding demonstrated across up to 165 samples in one study)	Moderate (sequencer + flow cell cost, decreasing with newer ONT platforms)	High sensitivity with direct methylation detection. No amplification-dependent LOD in the traditional sense.	Research stage	Promising for direct methylation profiling, but evidence for glioma-specific diagnostic application remains limited and requires clinical validation.
Enzyme-assisted recognition strategy	Uses methylation-sensitive enzymes or DNMT-based reactions	High specificity; signal amplification capability	Multi-step workflows; enzyme stability issues	Low–moderate	Low-Moderate	High specificity for targeted methylation analysis.	Research stage	Improves detection sensitivity for specific methylation sites
Immunoaffinity-based recognition strategy	Uses antibodies targeting 5-methylcytosine (5-mC)	High selectivity; simple workflow	Cross-reactivity; antibody variability	Moderate (ELISA-format allows batch processing)	Low	Moderate-to-high analytical sensitivity	Research stage	Useful for global methylation profiling in glioma samples

The table summarizes the detection principles, major advantages and limitations, analytical performance, current stage of clinical development, and potential applications of each biosensor platform. While electrochemical, optical, microfluidic, and nanopore-based transduction platforms, together with enzyme-assisted and immunoaffinity-based recognition strategies (shown in the final two rows as recognition chemistries that can be paired with those transducers rather than as standalone platforms), have demonstrated promising analytical performance for detecting glioma-associated DNA methylation, most remain in the research, laboratory validation, or early clinical evaluation stages and require further clinical validation before routine implementation in neuro-oncology.

### 4.1 Electrochemical biosensors

Because of their high sensitivity, affordability, and ease of miniaturization, electrochemical biosensors are among the most widely researched technologies for DNA methylation detection. These devices identify variations in electrical signals caused by interactions between recognition elements and methylation DNA, such as current, voltage, or impedance (37). Recent developments have focused on bisulfite-free detection techniques, such as direct electrochemical oxidation methods, immunoaffinity approaches that target 5-methylcytosine (5-mC), and enzyme-assisted methods (e.g., DNA methyltransferases and methylation-sensitive restriction enzymes) (35).

Furthermore, the use of nanomaterials such as graphene and gold nanoparticles has greatly increased signal transduction efficiency and sensitivity, enabling ultrasensitive detection of low-abundance methylated DNA targets. Because circulating tumor DNA (ctDNA) methylation indicators may be present at extremely low amounts in glioma applications, these characteristics are especially important (38, 39).

### 4.2 Optical biosensors

Optical biosensors utilize signal transduction mechanisms based on fluorescence, colorimetry, surface plasmon resonance (SPR), and surface-enhanced Raman scattering (SERS). These technologies enable highly sensitive, frequently label-free detection of DNA methylation events (40, 66).

While SPR-based techniques enable real-time monitoring of biomolecular interactions, fluorescence-based methods, such as Förster resonance energy transfer (FRET), enable the detection of methylation-specific hybridization (40, 66).

Moreover, nanomaterial-assisted optical biosensors improve detection sensitivity and signal amplification, making them well-suited for detecting minute epigenetic changes associated with tumor development. Their use in glioma biomarker detection is supported by these features, especially in multiplexed formats (41, 69, 70).

### 4.3 Microfluidic devices

The integration of biosensors with microfluidics is becoming increasingly popular to create smaller platforms with several advantages, including lower sample volume, shorter processing times, inexpensive analysis, and minimal reagent use. It is anticipated that integrating biosensors with microfluidic chips will improve analytical performance and expand the range of potential uses in clinical diagnostics. Recent advances in microfluidics have made it easier for researchers in industry and educational institutions to use these technologies for point-of-care (POC) diagnostics (42, 43).

Significantly, compact, fully integrated diagnostic systems can be made by combining microfluidic devices with optical or electrochemical detecting modules. Smartphone-assisted microfluidic devices, which allow for portable and easy-to-use DNA methylation detection, are examples of recent advancements (42, 44, 45). Such integration is particularly advantageous for glioma diagnostics, where rapid and minimally invasive testing (e.g., liquid biopsy) is essential.

### 4.4 Other emerging technologies

A revolutionary method for biomarker detection, nanopore-based sensing provides label-free, single-molecule analysis with remarkable sensitivity and specificity. These technologies offer direct insights into molecular size, shape, charge, and relationships by tracking ionic current modulations as individual molecules move through solid-state or biological nanopores. Recent developments in surface engineering, signal interpretation, and nanopore production have allowed them to operate in complex biological matrices and broaden their analytical scope from proteins and nucleic acids to peptides and tiny metabolites (46). Nanopore-based techniques are very promising for thorough glioma epigenetic profiling because they offer single-molecule resolution and enable simultaneous detection of DNA sequence and epigenetic alterations. In particular, the ability to detect clinically relevant methylation markers such as MGMT promoter methylation highlights the potential of these technologies for glioma diagnosis and treatment stratification. Nonetheless, the accuracy of nanopore methylation calling can vary across the MGMT promoter itself: within G-quadruplex-forming, G/C-rich stretches of the MGMT CpG island, single-molecule 5-methylcytosine detection has been reported to be less reliable than in flanking sequence, which underscores the need for improved basecalling models before nanopore assays are relied upon for quantitative MGMT calling (56).

### 4.5 Matching biosensor strategies to glioma methylation biomarkers

The selection of a biosensor platform should be influenced by the distinct attributes of the target methylation biomarker, such as its usual abundance, origin of the sample, and the sensitivity needed for making clinical decisions. **Table 3** aligns the biomarkers outlined in **Table 1** with the biosensor platforms discussed in Section 4, considering the sample type, target abundance, necessary analytical sensitivity, and clinical use. This framework aims to assist in selecting platforms for upcoming assay development, rather than suggesting that existing biosensor implementations have reached this level of glioma-specific validation. Generally, low-abundance liquid-biopsy targets focused on single loci (for example, GeLB, ctDNA-based MGMT) require highly sensitive, amplification-enhanced electrochemical or microfluidic platforms, while multi-locus or genome-wide signatures (G-CIMP, DMS, methylation class) are better paired with multiplexed optical or nanopore-based methods that emphasize breadth of coverage over single-molecule sensitivity.

**Table 3:** Biomarker-specific sample types, target abundance, sensitivity requirements, suitable biosensor platforms, and potential clinical applications for glioma-associated DNA methylation biomarkers.

Biomarker	Sample type	Typical target abundance	Required sensitivity	Best-suited platform(s)	Rationale	Clinical Context
MGMT promoter methylation	Tissue (fresh/FFPE); emerging ctDNA	Moderate in tissue; low in ctDNA	Moderate (tissue, ~1–5% methylated allele fraction detectable by MSP/pyrosequencing); high (ctDNA, femtomolar–attomolar)	Tissue: electrochemical, nanopore (FFPE-compatible). ctDNA: electrochemical with signal amplification, or enzyme-assisted	Tissue-level MGMT testing tolerates moderate LOD; nanopore’s single-molecule resolution and FFPE compatibility fit existing clinical workflows; ctDNA-based MGMT monitoring needs ultrasensitive amplification	Predictive (TMZ response); prognostic
G-CIMP (high/low)	Tissue	High (global hypermethylation signal across many CpG sites)	Moderate — signal is genome-wide, not single-locus	Optical (array-adjacent, multiplexed fluorescence); microfluidic panels	Global/multi-locus pattern doesn’t require single-molecule ultra-sensitivity; benefits more from multiplexing breadth than raw LOD	Diagnostic (IDH-mutant surrogate); prognostic
11-CpG IDH epi-signature	Tissue	Low-moderate (targeted CpG panel)	Moderate-high (needs to resolve small methylation differences at specific loci, incl. rare IDH1/2 variants)	Microfluidic multiplex electrochemical or optical arrays	Requires simultaneous, quantitative readout across ~11 defined loci — favors multiplexed chip-based formats over single-analyte platforms	Diagnostic (IDH classification)
GeLB score	Serum cfDNA	Low (cfDNA fraction, tumor-derived signal diluted in total cfDNA)	High (sub-picomolar to attomolar)	Electrochemical with nanomaterial/enzymatic amplification; microfluidic-integrated liquid biopsy platforms	Low-abundance serum signal demands ultrasensitive, amplification-based detection; microfluidic integration suits liquid-biopsy sample-handling needs	Diagnostic (liquid biopsy); monitoring (progression vs. pseudoprogression)
DNA methylation score (DMS)	Tissue	Moderate-high (multi-gene composite, ~5,700 genes)	Moderate (composite/array-derived, not single-molecule critical)	Optical/array-adjacent multiplexed platforms	Composite score derived from broad gene panel — favors high-multiplex optical or array-like readouts over ultra-high-sensitivity single-analyte sensors	Prognostic; predictive (immunotherapy response)
CMHR score (8-CpG)	Tissue	Moderate (defined 8-locus panel)	Moderate-high	Microfluidic multiplex electrochemical	Small, defined multi-locus panel — well suited to microfluidic multiplexed electrode arrays	Predictive (IDH status; immune infiltration correlate)
RTK1/RTK2/Mesenchymal methylation class	Tissue	High (genome-wide classification signal)	Moderate	Nanopore (genome-wide, single-molecule); array-adjacent optical	Whole-methylome class assignment is best served by platforms capable of broad genomic coverage rather than single-locus ultrasensitivity	Prognostic; clinical trial stratification

Selecting a platform also entails a multiplexing trade-off. Panels like the 11-CpG IDH signature or 8-CpG CMHR score necessitate concurrent multi-locus readout, which can be accomplished using electrochemical electrode arrays, spectrally multiplexed optical probes, or multi-channel microfluidic chips. However, multiplexing typically diminishes per-target sensitivity compared to single-analyte assays that attain the lowest reported LODs reported (e.g., the sub-attomolar/zeptomolar values in Section 6) (13, 16, 51, 71, 73). The right choice depends on the clinical question: a single highly sensitive marker (e.g., MGMT alone) versus a broader, lower-per-target-sensitivity panel (e.g., a combined MGMT/G-CIMP/GeLB).

## 5 Strategies for capturing and detecting methylated DNA sequences

Selective detection of methylated cytosines is essential for the development of methylation-specific biosensors. Common tactics include immunoaffinity techniques aimed at 5-mC, enzyme-assisted procedures, and hybridization-based detection (35). It is important to recognize that enzyme-assisted and immunoaffinity-based recognition are not independent transduction platforms; instead, they are molecular approaches that can be combined with any of the electrochemical, optical, nanopore or microfluidic transducers mentioned previously.

To distinguish between methylated and unmethylated DNA sequences, restriction endonuclease-based techniques and bisulfite conversion methods, both bisulfite-based and bisulfite-free, are also frequently employed. High specificity is made possible by these methods, which is crucial for identifying methylation markers linked to gliomas (35, 36, 47).

### 5.1 Integration with microfluidics for sample handling and analysis

Microfluidic biosensors use a collection of sensing elements on microfluidic chips to integrate detecting devices used in fluid infusion and control over signal reception, conversion, and presentation (48, 49). They incorporate microfluidic and biosensing technologies to accomplish the miniaturization, integration, and automation of disease diagnosis, and show high potential for application in the domains of cancer liquid biopsy and point-of-care testing (48). Moreover, microfluidics allows lower reagent consumption for quicker workflows and multiplexed detection of multiple biomarkers in a single device (50). The integration of advanced biosensors enables real-time detection of circulating tumor cells, nucleic acids, and exosomes, which supports novel applications in cancer diagnostics, POC testing, personalized medicine, and treatment monitoring (51). This is especially relevant in glioma, where combining various epigenetic indicators (such as MGMT promoter methylation) can improve diagnostic and prognostic accuracy (50, 52).

### 5.2 Pre-analytical considerations for liquid biopsy

The performance of biosensors for detecting ctDNA methylation is heavily reliant on pre-analytical handling, an aspect that has not been systematically explored for glioma applications. The type of collection tube affects the tolerance for pre-processing time: standard EDTA tubes necessitate plasma separation within a few hours to minimize leukocyte lysis and genomic DNA contamination of the cfDNA fraction, while cell-stabilizing tubes (e.g., Streck) prolong this timeframe but could modify subsequent methylation signals (53). Storage conditions, such as freeze-thaw cycles and duration before extraction, likewise influence cfDNA yield and fragmentation characteristics. Recent studies analyzing glioma tissue methylation data reveal that the tissue storage and sampling method, the sample preparation technique (such as sonication-based homogenization), and the particular 5-methylcytosine immunoprecipitation platform utilized allindependently influence MGMT methylation readings. This highlights that preanalytical variability is complicated by intratumoral heterogeneity and can significantly impact results even before the application of a detection platform (53). Extraction effectiveness and DNA fragmentation characteristics also influence the quality of assay input: significantly fragmented cfDNA, which is commonly found in apoptotic tumor-derived DNA, may decrease hybridization efficiency in probe-based biosensors or change translocation dynamics in nanopore systems (53).

### 5.3 Performance evaluation of glioma biosensors

Biosensors for DNA methylation detection have exhibited strong analytical performance, with excellent sensitivity and specificity thanks to enhanced recognition procedures and signal amplification approaches. Electrochemical and nanomaterial-enhanced platforms, in particular, can detect low-abundance targets over wide dynamic ranges, making them ideal for clinical applications with restricted sample availability (41, 54, 55, 57). Moreover, deep learning (DL) and artificial neural networks (ANNs) are gaining interest in biosensor research. Integrating machine learning into biosensors can eventually automate data extraction, processing, and analysis, which offers considerable benefits (58, 59).

### 5.4 Clinical validation studies using patient samples

Early identification and treatment are critical in cancer management, and the detection of circulating tumor DNA (ctDNA) has emerged as a promising approach for cancer screening and monitoring. As a result, liquid biopsy-based ctDNA analysis is gaining increasing attention in tumor diagnosis and therapy. However, conventional gene-detection methods, primarily based on polymerase chain reaction (PCR) and sequencing technologies, are limited by high cost, complexity, and lack of portability, making real-time and point-of-care ctDNA detection challenging. In this context, electrochemical biosensors have emerged as a promising alternative due to their high sensitivity, specificity, low cost, and ease of integration. Although current clinical applications of ctDNA detection still rely largely on PCR and sequencing approaches, biosensor-based platforms offer significant advantages, including improved accessibility and suitability for on-site and mobile clinical use. Several biosensor platforms have been tested using clinical materials, such as blood-derived circulating DNA and tumor tissues. These investigations show strong agreement with conventional methods, including PCR-based tests and sequencing methods, supporting their potential therapeutic value (35). In glioma, methylation biomarkers such as MGMT promoter methylation are well-established predictors of therapeutic response to temozolomide and overall prognosis, emphasizing the need for reliable and accessible detection platforms (55).

To date, the most substantive glioma-specific clinical validation of a methylation-detection approach comes from nanopore sequencing rather than from an electrochemical or optical biosensor. Halldorsson et al. evaluated MGMT promoter methylation by nanopore long-read sequencing across 165 central nervous system tumor samples, including 103 glioblastomas, and reported a strong correlation with pyrosequencing (R2 = 0.91) for the CpG sites measured by both methods, together with faithful reproduction of the MGMT-STP27 bead-array classification, while extending coverage to all 98 CpG sites of the MGMT CpG island (54). By contrast, electrochemical and optical platforms, despite their strong analytical performance in synthetic and non-glioma settings, have not yet been validated on glioma-specific clinical specimens. This lack of platform-level clinical validation for the electrochemical and optical biosensors reviewed here remains the central gap between their reported analytical sensitivity and any established clinical utility in glioma.

### 5.5 Comparison with traditional laboratory-based methods

Biosensors have several advantages over traditional approaches, including faster response time, greater portability, and lower cost. They are ideal for on-site quick screening and long-term dynamic monitoring, providing effective technological assistance for early detection and monitoring (60). However, despite substantial developments in biosensor technology for cancer diagnosis and prognosis, several challenges and limitations limit its implementation in clinical settings. Among these, sensitivity and specificity remain the most significant. Cancer biomarkers are often present at low concentrations, particularly in early stages, making it difficult for biosensors to achieve high sensitivity and specificity. In biological samples, such as blood and serum, other biomolecules can interfere with biosensor function (61).

Moreover, nonspecific binding or cross-reactivity can lead to false positives or negatives, thereby lowering the accuracy and reliability of test results (62). To solve this, ongoing work focuses on antifouling techniques on the biosensing surface, which are needed to limit nonspecific adsorption of biomolecules and improve biosensor performance (63). Addressing low biomarker levels in non-invasive samples frequently necessitates the use of novel enrichment approaches. Paper-based preconcentration or multiplexed lateral flow assays (LFAs) show promise for application in point-of-care settings. However, these systems require more refinement to achieve the reliability of laboratory techniques (61).

Signal amplification methods, including rolling circle amplification (RCA), catalytic hairpin assembly (CHA), and strand displacement reactions, are frequently used in biosensor platforms to improve sensitivity and detect low-abundance methylated DNA targets. A schematic overview of biosensor-based detection of DNA methylation in glioma, from sample collection to signal output, is shown in **Figure 3** (64, 65).

**Figure 3:**
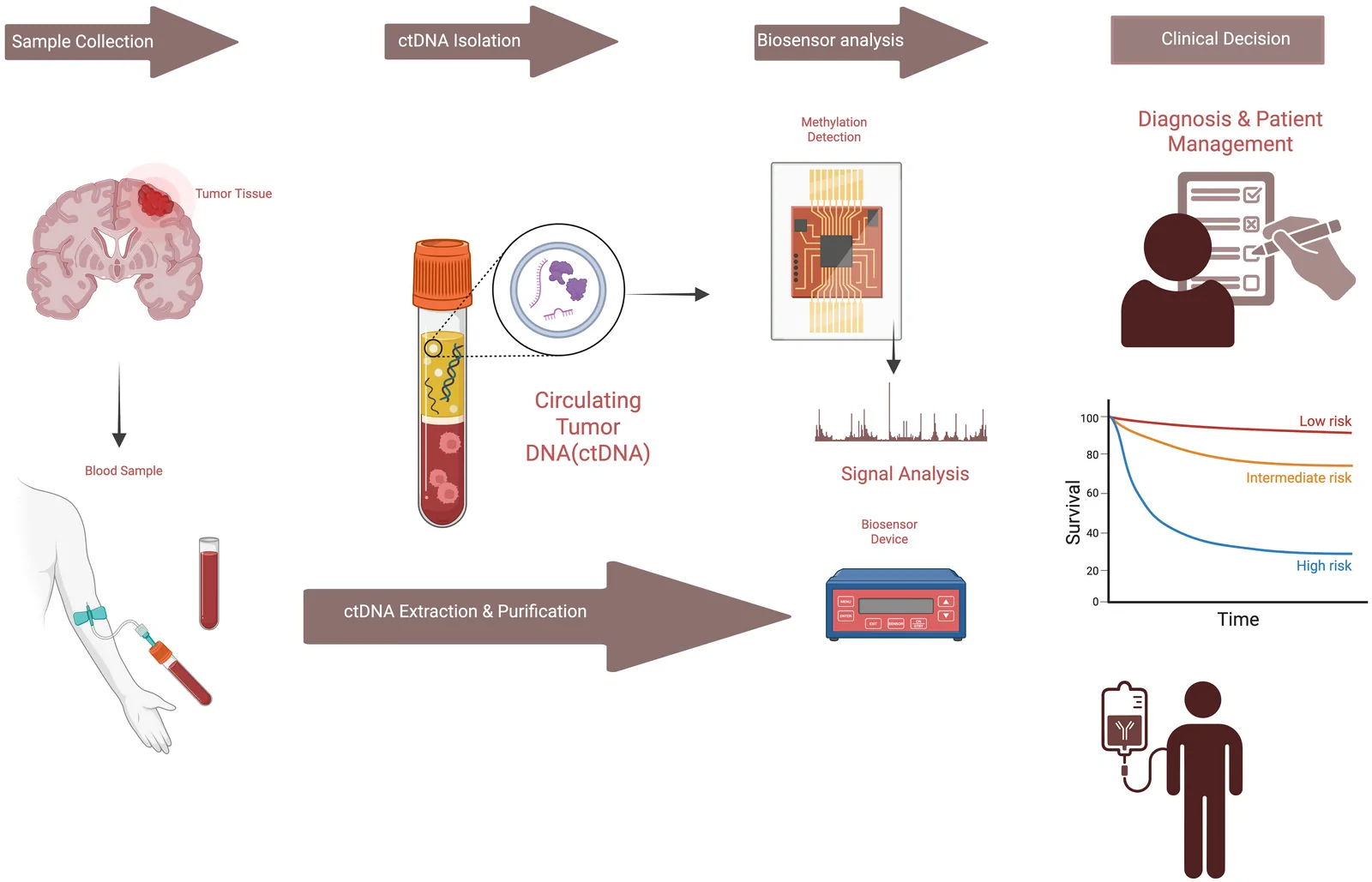
Workflow for biosensor-based detection of glioma-associated DNA methylation. Schematic representation of a general workflow from biological sample collection to methylation-associated signal detection and clinical interpretation. Tissue or liquid biopsy samples are collected, followed by blood processing and circulating tumor DNA (ctDNA) isolation and purification. Methylation-associated targets are subsequently analyzed using a biosensor platform, generating signals that can be processed to identify glioma-associated DNA methylation patterns. Depending on the assay design, methylation detection may incorporate methylation-specific recognition, enzymatic processing, amplification, or sequence-based approaches. The resulting signals can be analyzed to support diagnostic, prognostic, or treatment-monitoring applications. Selected components of the workflow may be adaptable to point-of-care (POC) testing; however, sample preparation, DNA extraction, and certain detection or amplification steps may remain laboratory-based. The workflow therefore illustrates the potential integration of biosensor technologies into glioma methylation analysis rather than implying that the complete workflow is currently POC-ready.

### 5.6 Clinical translation gap and practical considerations

Despite encouraging analytical performance, the translation of biosensor-based DNA methylation assays into routine neuro-oncology practice remains limited. Currently, clinically validated assessment of MGMT promoter methylation is primarily performed using laboratory-based techniques such as methylation-specific PCR, pyrosequencing, and methylation arrays, which require specialized equipment and trained personnel (55, 66). In comparison, biosensor platforms offer the potential for more rapid analysis, simplified workflows, portability, and point-of-care testing while requiring smaller sample volumes and reduced laboratory infrastructure (47, 60, 61). These features may also contribute to lower overall testing costs by reducing reagent consumption and instrumentation requirements, although comprehensive cost-effectiveness studies are still lacking (49, 61). Nevertheless, most biosensor platforms remain in the proof-of-concept or early clinical validation stage and have not yet been incorporated into routine clinical practice. Clinical translation also involves navigating regulatory and reimbursement routes that have not been addressed here. In the U.S., diagnostics typically need FDA 510(k) clearance, PMA approval, or CLIA (Clinical Laboratory Improvement Amendments)certification as a laboratory-developed test; in the EU, they are governed by the IVDR(*In Vitro* Diagnostic Regulation), which enforces rigorous clinical-evidence requirements. None of the platforms examined in this review have obtained FDA clearance or CE-IVD marking for the detection of glioma methylation. Despite receiving clearance, reimbursement (e.g., an assigned CPT code and payer coverage) is a separate, often slower hurdle (66). These regulatory and reimbursement barriers are likely to limit clinical adoption at least as much as remaining analytical challenges. Further large-scale validation, standardization, and regulatory evaluation will be required before these technologies can complement or replace existing molecular diagnostic methods for glioma (50, 55, 66).

## 6 Challenges and future directions

Currently, the clinical implementation of efficient glioma screening and molecular testing remains limited and varies according to tumor type and disease stage. Dysregulation of DNA methylation is associated with several pathological conditions, including cancers, neurodegenerative disorders, and aging. Tumor methylomes differ substantially from those of normal tissues, enabling discrimination between malignant and healthy cells based on tissue-specific DNA methylation patterns. These glioma-associated DNA methylation alterations can be detected in various body fluids using liquid biopsy approaches for diagnostic and prognostic assessment. Cell-free DNA (cfDNA) methylation screening delivers sufficient information, exploiting blood-based liquid biopsy methods. Three major categories of methods are used based on their mechanism of action: sodium bisulfite conversion, antibody-based detection of 5-methylcytosine (5-mC) (including enzyme-linked immunosorbent assay [ELISA]), and enzymatic cleavage using methylation-sensitive restriction enzymes. The ELISA method is widely used for the detection of DNA methylation. Several commercially available ELISA kits are available; however, these assays have poor sensitivity (66). Another challenging part of the analysis is the cross-reactivity with other co-existing biomolecules. To minimize cross-reacting biomolecules, the sample must be pre-processed. Therefore, the use of ELISA for methylated DNA detection for glioma diagnosis is not an appropriate method for clinical practice because it lacks specific biomarkers for glioma (67).

Although patients generally favor liquid biopsy for its non-invasive or minimally invasive nature, they face certain difficulties in methylated DNA detection, such as tumor heterogeneity and other exogenous factors. Cell type composition, technical issues, allele and strand-specific DNA methylation, cellular contamination, and accidental loss of methylated DNA in a specific site mainly cause tumor heterogeneity (68). Other issues, like fragmentation, low content, and other biomolecular influences in the signal, would influence the accuracy of the results. The methylation-sensitive restriction enzyme (MSRE) method is used to cleave unmethylated regions of CpG islands. The uncleaved methylated part will be used for the analysis. In the subsequent step, qPCR, bisulfite, or next-generation sequencing (NGS) is used. However, these are more complex and expensive.

Alternatively, biosensors are increasingly being developed as simple and miniaturized diagnostic platforms that can achieve very low analytical detection limits. These devices are intended to fulfill the ASSURED (Affordable, Sensitive, Specific, User-friendly, Rapid and Robust, Equipment-free, Deliverable) criteria for point-of-care testing (69). However, not all devices fully meet these criteria. Various biosensing methodologies, including fluorescence, colorimetry, surface plasmon resonance (SPR), Raman spectroscopy, surface-enhanced Raman spectroscopy (SERS), and electrochemistry, have been developed for the detection of epigenetically modified biomarkers. Multiple methylated DNA targets can be detected simultaneously using multicolor fluorescent biosensors. Different fluorophores with variable emission wavelengths can be labeled with probes complementary to target methylated DNA, while a broad-spectrum quencher such as cationic perylene diimide (PDI) can suppress fluorescence upon duplex formation with the target methylated DNA (70). The incorporation of nanomaterials into biosensors can further enhance analytical sensitivity. Adampourezare et al. reported an analytical range extending from 1 zM to 3 pM, with a reported LLOQ of 1 zM, using a synthetic methylated DNA target (71). However, such ultralow concentration values should be interpreted cautiously because they were obtained under controlled analytical conditions and do not necessarily represent the effective detection limit achievable in complex clinical specimens. At zeptomolar or sub-attomolar concentrations, the nominal concentration may correspond to fewer than one target molecule in a typical sample volume, making stochastic sampling, assay volume, background noise, and matrix effects important considerations. Therefore, these values should be regarded as idealized analytical performance measures rather than evidence of equivalent sample-level sensitivity in glioma liquid biopsy. Fluorescence-based approaches also have limitations, including autofluorescence from biological components, which can interfere with the fluorescence signal, and turbidity in blood or serum samples, which can scatter incident light and affect analytical accuracy (71).

Colorimetric analysis has unique advantages, including visual inspection of results by the naked eye, low cost, and ease of construction. Despite their qualitative or semiquantitative results, they are considered significant methods for rapid analysis that help make quick decisions for further treatments. Limitations include fading of color density over time, false-positive results, and non-specific interaction with other biomolecules. Nanomaterials are used to improve sensor sensitivity; however, the accumulation of nanomaterial complexes with complex biomolecules limits their application in real sample analysis (72). Electrochemical biosensors are more sensitive and allow multiplexed detection of several targets in a single measurement using screen-printed electrode arrays. Moreover, these miniaturized electrode systems are easy to transport and facilitate operation in limited-resource settings with minimal equipment requirements. For example, an electrochemical biosensor developed for methylated DNA detection by Hybridization Chain-Amplified Reaction, integrated with a Tetrahedral DNA nanostructure, required an electrochemical instrument. Though this method utilizes the instrument, it exhibits a LOD of 0.96 aM (73). As with the zeptomolar-range value above, this sub-attomolar LOD was achieved under regulated synthetic conditions and should not be considered equivalent to sample-level sensitivity in complex clinical matrices. The ideal method for developing biosensors for glioma will be rapid, low-cost, paper-based lateral flow assays that can be used regardless of geographical location or a nation’s economic status, as point-of-care testing. A smartphone-based fluorescence biosensor would be highly sensitive, avoiding the limitations of autofluorescence, sample damage, and light scattering by using near-infrared (NIR) fluorophores. We propose a paper-based lateral-flow assay that distinguishes methylated from unmethylated samples by the difference in fluorescence between test and control lines. In this device, a small amount of sample (<100 µL) would be enough to support methylation status determination within a few minutes.

## 7 Conclusion

DNA methylation is central to glioma biology and has emerged as an important biomarker for diagnosis, prognosis, and prediction of treatment response. Markers like MGMT promoter methylation have greatly aided patient classification and clinical decision-making. However, conventional detection technologies remain limited by high cost, complexity, and inaccessibility.

Biosensor technologies serve as an encouraging pre-clinical alternative, demonstrating strong analytical capability in proof-of-concept research; yet, their clinical usefulness in glioma is still unverified until extensive validation is conducted. The development of point-of-care diagnostic technologies is being driven by advancements in electrochemical, optical, and microfluidic platforms, as well as by the integration of nanomaterials and machine learning.

Despite these advancements, issues such as sensitivity in complex samples, standardization, and clinical validation persist. Addressing these constraints is crucial for integrating biosensor-based methylation detection into routine clinical practice. While previous reviews have largely focused on the biological and clinical significance of DNA methylation in glioma, this review additionally integrates current knowledge of glioma-associated methylation biomarkers with emerging biosensor technologies for their detection. By comparing electrochemical, optical, microfluidic, and nanopore-based biosensing platforms, together with enzyme-assisted and immunoaffinity-based recognition strategies that can be paired with them, we highlight their translational potential, current limitations, and future role in point-of-care molecular diagnostics. This integrated perspective provides a framework for bridging advances in epigenetic biomarker discovery with next-generation diagnostic technologies. Overall, the combination of DNA methylation biomarkers with novel biosensing technologies has the potential to significantly improve glioma diagnosis and patient care.
